# One-year weight-loss trajectories and associated baseline factors after laparoscopic sleeve gastrectomy: a retrospective cohort study

**DOI:** 10.1007/s13304-026-02863-9

**Published:** 2026-09-25

**Authors:** Yaxuan Wang, Rui Zhao, Anming Xu, Cao Li, Chunyan Lu, Jing Liao, Yanling Ma, Yue Wen

**Affiliations:** 1 Department of Gastrointestinal Surgery, West China Hospital, West China School of Nursing, Sichuan University, Sichuan University, Chengdu, China; 2 Department of Gastrointestinal Surgery, West China Hospital, West China School of Medicine, Sichuan University, Sichuan University, Chengdu, China; 3 Laboratory of Metabolism and Aging Research, National Clinical Research Center for Geriatrics and State key Laboratory of Respiratory Health and Multimorbidity, West China Hospital, Sichuan University, Chengdu, China

**Keywords:** Bariatric surgery, Laparoscopic sleeve gastrectomy, Weight-loss trajectory, Total weight loss, Visceral fat area, Retrospective cohort

## Abstract

This retrospective cohort study evaluated one-year weight-loss trajectories after laparoscopic sleeve gastrectomy and explored baseline factors associated with postoperative outcomes. Adult patients who underwent surgery between January 2021 and June 2024 were identified from an institutional bariatric surgery database. Body weight was assessed at 1, 3, 6, and 12 months after surgery. Generalized linear mixed-effects models were used to characterize longitudinal changes in body weight, body mass index, percentage total weight loss, and percentage excess weight loss, and multivariable linear regression was performed to identify baseline factors associated with percentage total weight loss at 12 months. A total of 489 patients were included, with a mean age of 32.5 ± 9.5 years and a mean body mass index of 36.7 ± 5.6 kg/m^2^. All weight-related indicators showed significant nonlinear trajectories characterized by rapid early change followed by gradual deceleration. At 12 months, mean percentage total weight loss was 30.2%, and 73.6% of patients achieved at least 20% total weight loss. Age, ethnicity, baseline body mass index, and visceral fat area were independently associated with postoperative weight-loss outcomes. These findings support trajectory-based monitoring after sleeve gastrectomy and may help identify patients at risk of suboptimal early weight loss.

## Introduction

Obesity has been demonstrated to be strongly associated with a wide range of comorbidities [1–5], including insulin resistance, type 2 diabetes mellitus(T2DM), hypertension, coronary artery disease, obstructive sleep apnea (OSA), polycystic ovary syndrome (PCOS), and impaired fertility. In addition to significantly increasing the mortality risk among individuals with T2DM and cardiovascular and cerebrovascular diseases [6], obesity is also positively associated with 13 types of malignancies [7].

With the rapid advancement of metabolic and bariatric surgery (MBS), its efficacy and safety in treating obesity and obesity-related metabolic disorders have been widely recognized. According to the 2023 annual report of the Chinese Obesity and Metabolic Surgery Database [8], approximately 37,000 bariatric procedures are performed in China each year, with sleeve gastrectomy (SG) accounting for 81.5% of all cases.

Although bariatric surgery provides significant clinical benefits, postoperative weight-loss outcomes are influenced by multiple factors, including preoperative weight, age, sex, obesity-related comorbidities, and surgical procedure [9, 10]. When postoperative weight loss falls short of patients’ expectations, frustration may develop and adversely affect adherence to dietary and behavioral recommendations [11, 12]. Long-term outcomes after sleeve gastrectomy may also be influenced by postoperative recovery, particularly when major complications compromise quality of life [13]. Regarding weight-loss response, previous studies have shown that early postoperative weight loss is strongly associated with the maximum weight reduction eventually achieved [14]. Therefore, accurately predicting and characterizing the trajectory of postoperative weight loss, as well as identifying its key determinants, is essential for establishing realistic preoperative expectations, improving clinician–patient communication, and enhancing postoperative adherence.

Although several predictive models have been proposed to estimate weight-loss outcomes after bariatric surgery, most focus on the magnitude of change in weight-related indicators and assume linear trajectories over time, failing to capture the nonlinear dynamics of postoperative weight loss [15]. Given the inherently complex and dynamic nature of the weight-loss process, traditional linear models often lack the ability to accurately reflect underlying physiological changes. To address this gap, the present study introduces a quadratic time component to quantitatively analyze postoperative changes in weight and BMI—both in terms of absolute values and rates of change. This approach aims to elucidate the nonlinear patterns of postoperative weight change and provide physiologically meaningful insights for improving the characterization of postoperative outcomes.

## Methods

### Study design and participants

This retrospective study included adult patients who underwent laparoscopic sleeve gastrectomy (LSG) at our hospital between January 2021 and June 2024. Baseline data were obtained from the hospital’s bariatric and metabolic surgery database and electronic medical records. Follow-up information, including body weight and postoperative recovery status, was collected at 1, 3, 6, and 12 months after surgery conducted by the research team. Follow-up availability was assessed independently at each time point; therefore, patients with missing data at an earlier visit could still contribute data at a later visit. The study was approved by the Ethics Committee of West China Hospital, Sichuan University (Approval No. 2025-1242) and written informed consent had been obtained from all patients at the time of surgery.

Inclusion criteria were as follows:

1. Age ≥ 18 years;
2. BMI ≥ 32.5 kg/m^2^;
3. BMI 27.5–32.5 kg/m^2^ with inadequate weight control after lifestyle modification and medical therapy, and fulfilling at least two components of metabolic syndrome;
4. A potential follow-up duration of at least 12 months after surgery and availability of at least 2 postoperative body-weight measurements during follow-up;
5. Willingness to participate and provision of informed consent.

Exclusion criteria were:

1. Pregnancy;
2. Undergoing revisional surgery or conversion to another bariatric procedure after LSG;
3. Pregnancy during postoperative follow-up.

### Sleeve gastrectomy

All procedures were performed under general anesthesia using a standard four-port laparoscopic technique. The greater curvature was mobilized beginning approximately 4 cm from the pylorus and extending cephalad to the angle of His. A 36-Fr bougie was inserted along the lesser curvature to guide gastric division. Sleeve creation was completed with sequential stapling toward the gastroesophageal junction, ensuring complete resection of the fundus. The staple line was reinforced with oversewing of the seromuscular layer, and hemostasis was confirmed prior to trocar-site closure.

### Variables considered

The following variables were collected for all enrolled patients: age, sex, educational level, ethnicity, and other general demographic characteristics. Clinical measurements at baseline included height, weight, waist circumference, hip circumference, subcutaneous fat area, visceral fat area, blood pressure, blood glucose, and length of hospital stay. Visceral obesity was defined as a visceral fat area (VFA) ≥ 100 cm^3^ [16]. Hypertension was defined as systolic blood pressure ≥ 140 mmHg, diastolic blood pressure ≥ 90 mmHg, or current use of antihypertensive medication [17]. T2DM was diagnosed according to the 2023 American Diabetes Association criteria [18]. Insulin resistance was assessed using the HOMA-IR index, with HOMA-IR > 2 indicating insulin resistance [19, 20]. The overall clinical assessment and surgical management were conducted in accordance with contemporary clinical practice guidance for bariatric and metabolic surgery [21].

Postoperative weight-loss outcomes were evaluated and predicted using body weight, BMI, percentage of total weight loss (TWL%), and percentage of excess weight loss (EWL%). TWL% was calculated as (baseline weight − current weight)/baseline weight × 100%, with TWL% ≥ 20% considered a favorable outcome. EWL% was calculated as (baseline weight − current weight)/(baseline weight − ideal weight) × 100%, where ideal weight was defined based on a BMI of 23 kg/m^2^ [22–24]; EWL% ≥ 50% was considered indicative of a good outcome.

### Statistical analysis

Continuous variables were described as means and standard deviations, whereas categorical variables were summarized as frequencies and percentages. A mixed-effects models was applied to evaluate longitudinal postoperative weight-loss outcomes. This model appropriately accounts for the non-independence of repeated measurements and provides unbiased parameter estimates under the assumption of missing at random (MAR) using maximum likelihood estimation. By incorporating subject-specific random intercepts, the model estimates the overall temporal trend while quantifying and controlling for baseline heterogeneity across individuals, thereby improving statistical efficiency [25, 26].

Body weight, BMI, TWL%, and EWL% were selected as outcome indicators for weight-loss evaluation. Body weight and BMI analyses included measurements at baseline and at 1, 3, 6, and 12 months postoperatively, whereas TWL% and EWL% analyses used data from the four postoperative follow-up time points. The fixed-effects component of the model included both linear and quadratic terms for time to capture potential nonlinear trajectories of weight loss. The random-effects component included a subject-level random intercept.

To assess model fit, the Akaike Information Criterion (AIC), intraclass correlation coefficient (ICC), and R^2^ were calculated. Lower AIC values indicate better model fit; differences greater than 10 between the quadratic and linear models were considered strong evidence supporting the more complex model. The ICC quantified the proportion of total variance attributable to between-subject differences; higher ICC values indicate stronger within-subject correlation and greater necessity for mixed-effects modeling. The conditional R^2^ reflected the total variance explained by both fixed and random effects, while the marginal R^2^ represented variance explained solely by fixed effects.

Finally, linear regression was performed to evaluate factors associated with weight-loss outcomes, with baseline characteristics as independent variables and TWL% as the dependent variable. All analyses were conducted using R software (version 4.2.0). Two-sided tests were adopted, with a significance level set at 0.05.

## Results

### Baseline characteristics of the study population

A total of 489 patients who underwent sleeve gastrectomy were included in this study, with a mean age of 32.5 ± 9.5 years. Among them, 319 were female (65.24%) and 170 were male (34.76%). Most patients were of Han ethnicity (376 cases, 76.89%), while 113 (23.11%) were from ethnic minority groups. Regarding educational level, 263 patients (55.14%) had education below the undergraduate level, whereas 214 patients (44.86%) had an undergraduate degree or higher.

At baseline, the mean body weight was 101.6 ± 20.4 kg, and the mean BMI was 36.7 ± 5.6 kg/m^2^. The mean waist circumference was 114.4 ± 5.0 cm, and the mean hip circumference was 115.6 ± 9.4 cm. The average VFA was 203.6 ± 44.4 cm^3^. Insulin resistance was present in 423 patients (93.58%). A total of 147 patients (31.55%)—nearly one-third—had T2DM. Elevated blood pressure was observed in 114 patients (24.84%). The mean length of hospital stay was 7.7 ± 2.0 days (Table 1).

**Table 1:** Demographic and perioperative characteristics of the study population

Variables	Value (*n* = 489)
Age (y)	32.5 ± 9.5
*Gender*	
Male	170 (34.76)
Female	319 (65.24)
*Ethnicity*	
Han	376 (76.89)
Ethnic minority	113 (23.11)
*Degree*	
Below Bachelor’s degree	263 (55.14)
Bachelor’s degree or above/higher	214 (44.86)
Weight (kg)	101.6 ± 20.4
BMI (kg/m^2^)	36.7 ± 5.6
Waist (cm)	114.4 ± 5.0
Hip (cm)	115.6 ± 9.4
VFA (cm^3^)	203.6 ± 44.4
Length of hospital stay (days)	7.7 ± 2.0
*Hypertension*	
Yes	114 (24.84)
No	345 (75.16)
*T2DM*	
Yes	147 (31.55)
No	319 (68.45)
*Insulin resistance*	
Yes	423(93.58)
No	29 (6.42)

Data are presented as mean ± standard deviation for continuous variables and number (percentage) for categorical variables

BMI, body mass index; VFA, visceral fat area; T2DM, type 2 diabetes mellitus

### Follow-up completion

Among the 489 included patients, follow-up data were available for 378 patients (77.3%) at 1 month, 366 (74.8%) at 3 months, 356 (72.8%) at 6 months, and 406 (83.0%) at 12 months after surgery. The higher follow-up rate at 12 months was likely related to intensified follow-up efforts at the final assessment, including repeated telephone contact and reminders for postoperative review.

### Evaluation of weight-loss outcomes and trajectory modeling

Table 2 shows a high concordance between observed and model-predicted values for weight, BMI, TWL%, and EWL% at all follow-up time points, indicating good model fit. Parameter estimates from the mixed-effects models are summarized in Table 3. All four outcomes demonstrated significant nonlinear (quadratic) trajectories over time, with both linear and quadratic terms reaching statistical significance (all *P* < 0.001).

**Fig. 1:**
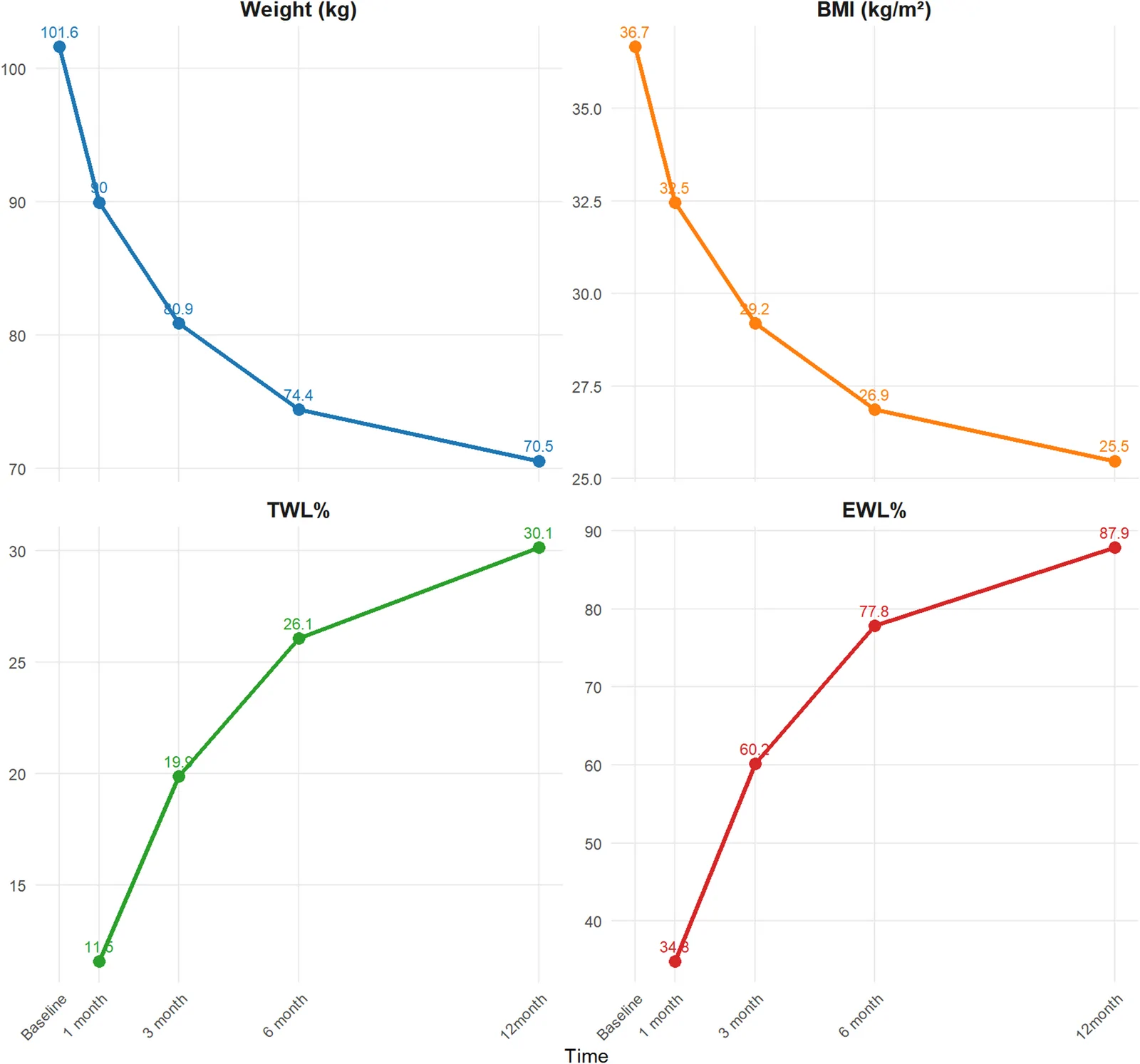
Model-predicted trajectories of postoperative weight-related outcomes over time. Model-predicted trends of body weight (kg), body mass index (BMI, kg/m^2^), percentage of total weight loss (TWL%), and percentage of excess weight loss (EWL%) from baseline to 12 months after sleeve gastrectomy are shown. Predicted values were derived from mixed-effects models incorporating both linear and quadratic time effects. The trajectories demonstrate a nonlinear pattern characterized by rapid early changes followed by gradual deceleration

Mean body weight decreased from 101.6 ± 20.4 kg at baseline to 70.2 ± 14.2 kg at 12 months (– 31.3 ± 12.9 kg). Model estimates showed a significant linear decline (β = − 15.57, SE = 0.85) and a quadratic component (*β* = 1.30, SE = 0.14; both *P* < 0.001), consistent with a rapid early decrease followed by gradual deceleration (Fig. 1). The ICC was 0.842, indicating substantial between-individual variability. Similarly, mean BMI decreased from 36.7 ± 5.6 to 25.4 ± 3.9 kg/m^2^ (– 11.3 ± 4.3 kg/m^2^), with significant linear and quadratic terms (both *P* < 0.001) and an ICC of 0.766.

At 6 months, mean TWL% reached 26.0%, and increased to 30.2% at 12 months, with 73.6% of patients achieving TWL% ≥20%. TWL% followed a significant quadratic increasing trajectory (linear β = 13.62; quadratic *β* = − 1.06; both *P* < 0.001), with an ICC of 0.409. Mean EWL% increased from 61.1% at 3 months to 88.2% at 12 months, and 77.3% of patients achieved EWL% ≥50%. EWL% also demonstrated a robust nonlinear pattern (linear *β* = 44.51; quadratic *β* = − 3.83; both *P* < 0.001), with moderate between-individual variability (ICC = 0.668).

All four outcomes demonstrated statistically significant linear trends, establishing the feasibility of incorporating quadratic terms. Model fit indices showed that AIC differences between quadratic and linear models exceeded 10 for all outcomes, and likelihood ratio tests were significant (*P* < 0.001), confirming the nonlinear (quadratic) nature of postoperative weight change and the superiority of the quadratic model. The conditional R^2^ values for all models ranged from 0.763 to 0.899, indicating excellent explanatory power.

**Table 2:** Observed and model-predicted values of weight-related outcomes at baseline and during follow-up

Variables	Baseline		1 m		3 m		6 m		12 m	
	Measurement value	Predicted value	Measurement value	Predicted value	Measurement value	Predicted value	Measurement value	Predicted value	Measurement value	Predicted value
Weight	101.6 ± 20.4	101.6	89.8 ± 17.3	90.0	78.5 ± 14.7	80.9	73.0 ± 17.1	74.4	70.2 ± 14.2	70.5
BMI	36.7 ± 5.6	36.7	32.5 ± 5.0	32.5	28.6 ± 4.1	29.2	26.6 ± 4.6	26.9	25.4 ± 3.9	25.5
TWL %			11.5 ± 3.5	11.6	19.7 ± 4.4	19.9	26.0 ± 6.9	26.1	30.2 ± 8.6	30.1
EWL %			34.3 ± 14.3	34.8	61.1 ± 20.1	60.2	80.3 ± 26.1	77.8	88.2 ± 28.5	87.9

The table presents observed measurements and corresponding model-predicted values for body weight, body mass index (BMI), percentage of total weight loss (TWL%), and percentage of excess weight loss (EWL%) at baseline and at 1, 3, 6, and 12 months after surgery. Observed values are expressed as mean ± standard deviation

BMI, body mass index; TWL%, percentage of total weight loss; EWL%, percentage of excess weight loss

**Table 3:** Results of mixed-effects models for postoperative weight-related outcomes

Variables	Intercept, β (SE)	Linear, β (SE)	Quadratic, β (SE)	ICC	Marginal *R*^2^/ conditional *R*^2^	AIC difference
Weight	115.89 (1.17)***	− 15.57 (0.85)***	1.30 (0.14)***	0.842	0.359/0.899	79.2
BMI	41.82 (0.36)***	− 5.62 (0.28)***	0.47 (0.05)***	0.766	0.489/0.800	91.3
TWL%	− 11.45 (2.84)***	13.62 (1.86)***	− 1.06 (0.26)***	0.409	0.599/0.763	13.0
EWL%	− 38.91 (8.09)***	44.51 (5.30)***	− 3.83 (0.75)***	0.668	0.487/0.829	24.8

The table presents fixed-effect parameter estimates for generalized linear mixed-effects models assessing postoperative trajectories of body weight, body mass index (BMI), percentage of total weight loss (TWL%), and percentage of excess weight loss (EWL%)

Estimates are reported as regression coefficients (β) with standard errors (SE). Intraclass correlation coefficients (ICC) represent the proportion of total variance attributable to between-individual differences. Marginal R² reflects variance explained by fixed effects alone, whereas conditional R² reflects variance explained by both fixed and random effects. AIC difference represents the difference between linear and quadratic models

BMI, body mass index; TWL%, percentage of total weight loss; EWL%, percentage of excess weight loss; ICC, intraclass correlation coefficient; AIC, akaike information criterion. ***P < 0.001

### Factors associated with TWL%

In univariate analyses, age, ethnicity, educational level, baseline BMI, and visceral fat area (VFA) were associated with postoperative TWL% (Table 4). Variables with *P* < 0.2 in univariate analyses were entered into the multivariable linear regression model. After confirming the absence of multicollinearity, age, ethnicity, baseline BMI, and VFA remained independently associated with postoperative TWL%. TWL% decreased with increasing age (*β* = − 0.152, 95% CI − 0.260 to − 0.044, *P* < 0.01). Compared with Han patients, individuals from ethnic minority groups achieved higher TWL% (*β* = 2.183, 95% CI 0.098–4.267, *P* < 0.05). Higher baseline BMI was associated with greater TWL% (*β* = 0.375, 95% CI 0.153–0.597, *P* < 0.01), and higher baseline VFA also predicted greater TWL% (*β* = 0.032, 95% CI 0.004–0.061, *P* < 0.05).

**Table 4:** Univariate and multivariable analyses of factors associated with percentage of total weight loss (TWL%) at 12 months after sleeve gastrectomy

Baseline variables	Univariate		Multivariable	
	OR (95% CI)	*P*	OR (95% CI)	*P*
Age (y)	− 0.208 (− 0.301 to − 0.116)	< 0.001	− 0.152 (− 0.260 to − 0.044)	**0.006**
*Gender*				
Male	1.00 (Ref)			
Female	− 0.373 (− 2.139 to 1.394)	0.678		
*Ethnicity*				
Han	1.00 (Ref)		1.00 (Ref)	
Ethnic Minority	2.230 (0.312–4.148)	**0.023**	2.183 (0.098–4.267)	**0.040**
*Degree*				
Below Bachelor’s degree	1.00 (Ref)		1.00 (Ref)	
Bachelor’s degree or above/higher	1.941 (0.023–3.649)	**0.026**	1.251 (− 0.568 to 3.069)	0.177
BMI (kg/m^2^)	0.623 (0.486–0.761)	< 0.001	0.375 (0.153–0.597)	**0.001**
VFA (cm^3^)	16.435 (12.174–20.696)	< 0.001	0.032 (0.004–0.061)	**0.025**
Length of hospital stay (days)	0.349 (− 0.082 to 0.780)	0.112	0.074 (− 0.410 to 0.577)	0.764
*Hypertension*				
No	1.00 (Ref)			
Yes	0.432 (− 1.560 to 2.424)	0.670		
*T2DM*				
No	1.00 (Ref)			
Yes	− 0.376 (− 2.289 to 1.537)	0.699		
*Insulin resistance*				
No	1.00 (Ref)			
Yes	0.700 (− 2.693 to 4.094)	0.685		

Univariate and multivariable linear regression analyses were performed to identify baseline factors associated with postoperative TWL%. Variables with *P* < 0.20 in univariate analysis were included in the multivariable model. Results are presented as regression coefficients with 95% confidence intervals (CI). Adjusted R^2^ for the multivariable model was 0.216. Bolded P values indicate statistical significance (P < 0.05).

BMI, body mass index; VFA, visceral fat area; T2DM, type 2 diabetes mellitus; Ref, reference category

## Discussion

Using data from 489 patients, we examined one-year weight-loss trajectories after sleeve gastrectomy using mixed-effects models. Weight, BMI, TWL%, and EWL% all showed a nonlinear pattern, with rapid changes during the first six months followed by a slower rate of weight loss. This pattern is consistent with previous studies showing that weight loss is greatest during the early postoperative period and gradually stabilizes toward 12 months [14, 27]. Early weight change is clinically important because an inadequate initial response may be associated with poorer long-term outcomes [28]. Our findings therefore highlight the importance of monitoring weight-loss trajectories during the first six months after surgery. At 12 months, mean TWL% and EWL% were 30.2% and 88.2%, respectively. These results were similar to those reported by Xu et al. in Chinese patients, with a TWL% of 28.24% and an EWL% of 83.44% [27]. In addition, 77.3% of patients achieved EWL% ≥ 50%, compared with 82.9% in the study by Albanopoulos et al. [29]. The significant quadratic time effects confirmed that the rate of weight change decreased over time. The relatively high ICC values for weight and BMI also indicated considerable differences between patients, supporting the use of mixed-effects models for longitudinal analysis and the need for individualized postoperative follow-up.

Previous studies have identified several preoperative factors associated with postoperative weight loss, including age, BMI, sex, T2DM, and psychological status. However, their effects remain inconsistent across studies because of differences in patient characteristics and surgical procedures [30]. We used TWL% as the primary outcome because it is independent of ideal body weight and may provide a more reliable measure of postoperative weight loss than EWL% [31, 32].

In our analysis, older age was associated with lower TWL%, whereas higher baseline BMI was associated with greater TWL%. Similar associations have been reported previously, although the evidence remains inconsistent [30, 35–39]. Differences in patient characteristics, surgical procedures, follow-up duration, and weight-loss definitions may partly explain these discrepancies. Therefore, age and BMI should be considered baseline factors associated with postoperative weight loss rather than direct determinants of treatment response [28, 33–37].

Patients from ethnic minority groups achieved a 2.18-percentage-point higher TWL% than Han patients. This finding is noteworthy because evidence comparing postoperative weight loss between Han and ethnic minority populations in China remains limited. Han and ethnic minority populations may differ in culturally shaped dietary habits and lifestyle patterns, including staple-food choices, meal composition, eating practices, and levels of daily physical activity. These differences may influence postoperative dietary adaptation, adherence to lifestyle recommendations, and subsequent weight loss. Further prospective studies incorporating detailed assessments of dietary intake, lifestyle behaviors, postoperative adherence, and metabolic characteristics are needed to clarify the factors underlying this association.

An important strength of this study was the inclusion of anthropometric and body-fat distribution measures in addition to BMI. Waist- and hip-related measurements reflect central fat distribution, whereas VFA, assessed by abdominal CT, provides a direct and objective measure of visceral adiposity. In our analysis, higher baseline VFA was independently associated with greater TWL%. Previous studies have also linked visceral adiposity with postoperative complications and weight-loss outcomes after bariatric surgery [38–40]. These findings suggest that CT-derived VFA may provide clinically relevant information complementary to BMI when assessing obesity severity and postoperative response. The inclusion of VFA may therefore improve the preoperative characterization of patients and help explain individual differences in weight-loss outcomes after sleeve gastrectomy.

As this was a single-center study, further validation in other clinical settings would strengthen the broader applicability of our findings. Follow-up data were primarily obtained during hospital visits, and telephone contact with patients who did not return had a relatively low response rate, resulting in missing observations at some time points. Although mixed-effects models allowed the use of all available longitudinal data, incomplete follow-up may still have influenced the estimates. In addition, the one-year follow-up did not capture long-term weight maintenance or weight regain. Nevertheless, the model provides a practical framework for estimating postoperative weight-loss trajectories and may help identify patients whose weight loss deviates from the expected pattern.

## Conclusion

Sleeve gastrectomy produced significant early weight loss that followed a nonlinear “rapid-then-slow” trajectory. Age, ethnicity, BMI, and visceral fat area independently influenced TWL%, reflecting substantial individual variability. These findings highlight the value of trajectory-based monitoring for early identification of suboptimal responders.
